# Dectin-2-dependent host defense in mice infected with serotype 3 *Streptococcus pneumoniae*

**DOI:** 10.1186/s12865-015-0139-3

**Published:** 2016-01-05

**Authors:** Yukiko Akahori, Tomomitsu Miyasaka, Masahiko Toyama, Ikumi Matsumoto, Anna Miyahara, Tong Zong, Keiko Ishii, Yuki Kinjo, Yoshitsugu Miyazaki, Shinobu Saijo, Yoichiro Iwakura, Kazuyoshi Kawakami

**Affiliations:** Department of Medical Microbiology, Mycology and Immunology, Tohoku University Graduate School of Medicine, Miyagi, Japan; Department of Chemotherapy and Mycoses, National Institute of Infectious Diseases, Tokyo, Japan; Division of Molecular Immunology, Medical Mycology Research Center, Chiba University, Chiba, Japan; Center for Animal Disease Models, Research Institute for Biomedical Sciences, Tokyo University of Science, Chiba, Japan; Present address: Japanese Red Cross Society, Tokyo, Japan; Present address: Department of Pathophysiology, Tohoku Pharmaceutical University, Miyagi, Japan; Present address: Ibaraki Prefectural Hospital, Ibaraki, Japan

**Keywords:** *Streptococcus pneumonia*, Dectin-2, Neutrophils, Anti-capsular polysaccharide IgG, IFN-γ

## Abstract

**Background:**

*Streptococcus pneumoniae*, a major causative bacterial pathogen of community-acquired pneumonia, possesses a thick polysaccharide capsule. Host defense against this bacterium is mediated by activation of innate immune cells that sense bacterial components. Recently, C-type lectin receptors (CLRs) have garnered much attention in elucidating the recognition mechanism of pathogen-derived polysaccharides.

**Methods:**

In the present study, we first compared the clinical course and neutrophil accumulation in the lungs of Dectin-2 knock-out (KO) and wild type (WT) mice. Mice were infected intratracheally with a serotype 3 strain of *S. pneumoniae*, and *S. pneumoniae* bacterial engulfment by neutrophils and inflammatory cytokine and anti-pneumococcal polysaccharide-specific IgG levels were evaluated in bronchoalveolar lavage fluid (BALF). We also examined the effect of Dectin-2 deficiency on interleukin (IL)-12 production by bone marrow-derived dendritic cells (BM-DCs) stimulated with the bacterial components.

**Results:**

*S. pneumonia-*infected Dectin-2KO mice had a shorter survival time, larger bacterial burden and lower interferon gamma (IFN-γ) production in the lungs than WT mice. Although neutrophilic infiltration in the lungs was equivalent between Dectin-2KO mice and WT mice, *S. pneumonia* engulfment by neutrophils was attenuated in Dectin-2KO mice compared to WT mice. The anti-pneumococcal polysaccharide-specific IgG and IgG3 levels in BALF were lower in Dectin-2KO mice than in WT mice. When BM-DCs were stimulated with *S. pneumoniae* culture supernatant or its Concanavalin A (ConA)-bound fraction, IL-12 production was abrogated in Dectin-2KO mice compared to WT mice.

**Conclusions:**

We demonstrated that Dectin-2 is intimately involved in the host defense against infection with a serotype 3 strain of *S. pneumoniae*. Dectin-2-dependent IL-12 production may contribute to IFN-γ synthesis and subsequent production of serotype-specific anti-capsular polysaccharide IgG after *S. pneumoniae* infection, which may promote *S. pneumoniae* bacterial opsonization for engulfment.

## Background

*Streptococcus pneumoniae* is recognized as a major bacterial agent that causes community-acquired pneumonia and other invasive diseases, such as bacteremia and meningitis [1, 2]. Upon infection with *S. pneumoniae*, the innate mechanism of the early phase of immunity plays an important role in host defense, which is largely mediated by neutrophil-dependent immune responses. The infiltration of many neutrophils into the alveolar spaces eradicates the infection via an immunoglobulin G (IgG)-mediated opsonophagocytic killing (OPK) mechanism and the production of reactive oxygen species (ROS) [3]. IgG3 that is specific for pneumococcal capsular polysaccharide, a thymus-independent type 2 (TI-2) antigen, is highly protective against infection with *S. pneumoniae* [4]. Recently, we reported similar findings that pneumococcal polysaccharide vaccine (PPV) immunization increases serotype 3-specific IgG3 serum levels, which facilitates survival after pneumococcal infection [5]. CXC chemokines, including macrophage inflammatory protein (MIP)-2 and keratinocyte-derived chemokine (KC), a homologue of human interleukin (IL)-8, were involved in neutrophil accumulation at the inflammatory sites. Previous studies showed that death in mice challenged with *S. pneumoniae* was preceded by bacterial growth within 2 days after infection and was associated with a delayed increase in pulmonary MIP-2 levels and neutrophil recruitment [6].

DC-associated C-type lectin-2 (Dectin-2), a C-type lectin receptor, possesses a carbohydrate recognition domain for the Ca^2+^-dependent recognition of mannose oligosaccharides [7, 8]. Dectin-2 invokes innate immune responses and subsequent adaptive immunity in fungal infection. Dectin-2-mediated recognition of *Candida albicans* leads to NF-κB activation, which induces IL-1β, IL-12 and IL-23 production by macrophages [7]. IL-1β and IL-23 potentiate neutrophil recruitment into the infected tissues through inducing IL-17A production [9–12]. T helper (Th)-1 cell-related cytokines, IL-12 and interferon gamma (IFN-γ), play a critical role in the neutrophil-mediated host defense against *S. pneumoniae* infection, which is correlated with MIP-2 and TNF-α production [13, 14]. Mice with targeted disruption of the IL-12 or IFN-γ gene are highly susceptible to pneumococcal pneumonia [13, 15].

In the present study, we aimed to determine the role of Dectin-2 in the neutrophil-mediated host defense to *S. pneumoniae* infection using mice with a genetic disruption of Dectin-2. We found that Dectin-2 knock out (KO) mice were more susceptible to this infection than wild type (WT) mice, and our results suggest that Dectin-2-dependent IL-12 production may contribute to IFN-γ synthesis and subsequent production of serotype-specific anti-capsular polysaccharide IgG after *S. pneumoniae* infection, which may promote opsonization of this bacterium for engulfment.

## Results

### Role of Dectin-2 in the host defense to pneumococcal infection

**Fig. 1:**
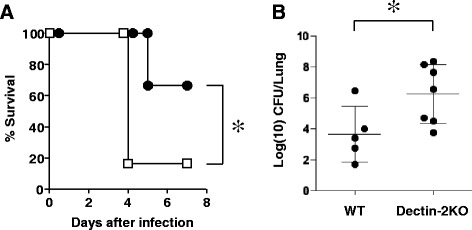
Effect of Dectin-2 deficiency on *S. pneumoniae* infection. WT mice and Dectin-2KO mice were infected with *S. pneumoniae*. **a** The number of live mice was recorded daily. Closed circles, WT mice (*n* = 6); open squares, Dectin-2KO mice (*n* = 6). **b** Bacterial load in the lungs was examined on day 3 post-infection. Each symbol shows each mouse, and the bars indicate the mean ± SD. Similar results were obtained in three independent experiments. *p < 0.05

To clarify whether Dectin-2 deficiency affects early-phase host protection against pneumococcal infection, we initially examined the susceptibility of Dectin-2KO mice to *S. pneumoniae* infection and compared it with WT mice by recording the survival rate of the infected mice and also the bacterial load in their lungs. Dectin-2KO mice had a lower survival rate (17 % by day 4 after intratracheal *S. pneumoniae* infection), whereas 67 % of WT mice survived throughout the observation period (Fig. 1a). The difference in the survival rate was statistically significant. In addition, the number of live bacterial colonies was significantly lower in the lungs of WT mice than in Dectin-2KO mice on day 3 post-infection (Fig. 1b). These data indicate that Dectin-2 plays a critical role in early-phase host defense against pneumococcal infection.

### Role of Dectin-2 in the neutrophil-mediated host defense to pneumococcal infection

**Fig. 2:**
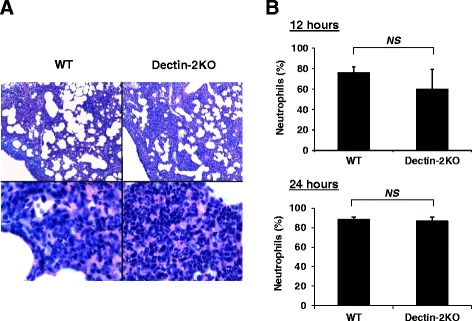
Equivalent level of neutrophil infiltration in lungs between WT and Dectin-2KO mice. WT mice and Dectin-2KO mice were infected with *S. pneumoniae.* **a** The lung section obtained 12 h post-infection was stained with hematoxylin-eosin (H-E), and then observed using light microscopy. Original magnifications: ×100 and × 1000. **b** Cells in BALF at 12 h and 24 h post-infection were stained with Diff-Quick, and the composition of neutrophils was then quantified. Each group consists of five to seven mice. Similar results were obtained in three independent experiments. *NS*, not significantly different

Neutrophils rapidly accumulate at the most infected sites and they play a central role in eradicating bacteria after pulmonary *S. pneumoniae* infection [16]. Therefore, to address the role of Dectin-2 in neutrophil-mediated host defense against this bacterial pathogen, we first evaluated neutrophil recruitment in the infected lungs. A histological analysis showed no apparent difference in inflammatory cell infiltration in the lungs between WT and Dectin-2KO mice 12 h after infection with *S. pneumoniae*. When observed at a higher magnification, neutrophil accumulation in the alveolar spaces was shown to be comparable between these mice (Fig. 2a). In addition, the number of neutrophils in bronchoalveolar lavage fluid (BALF) was almost equivalent between WT and Dectin-2KO mice 12 h and 24 h after infection (Fig. 2b).

**Fig. 3:**
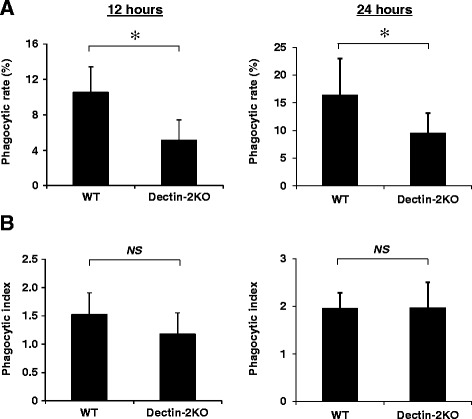
Effect of Dectin-2 deficiency on the engulfment of pneumococcus by neutrophils. WT mice and Dectin-2KO mice were infected with *S. pneumoniae.* The phagocytic rate (**a**) and phagocytic index (**b**) of neutrophils in BALF were calculated 12 h and 24 h after infection. The number of neutrophils was estimated by multiplying the total cell count by its proportion identified in morphological analysis. Each group consists of five to seven mice. Similar results were obtained in three independent experiments. *, *p < 0.05*; *NS*, not significantly different

To further clarify the role of Dectin-2 in the neutrophil-mediated host defense, we evaluated the phagocytosis rate and phagocytosis index of these cells in BALF 12 h and 24 h after infection. As shown in Fig. 3a, the rate of neutrophils engulfing pneumococcus was significantly lower in Dectin-2KO mice than in WT mice at both time points, although the average number of pneumococcus per engulfed neutrophil did not differ largely between these mice (Fig. 3b).

### Role of Dectin-2 in the production of proinflammatory cytokines and chemokines after pneumococcal infection

**Fig. 4:**
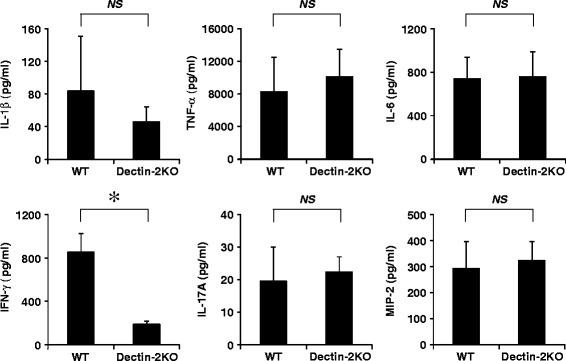
Effect of Dectin-2 deficiency on cytokine production in the lungs after pneumococcal infection. WT mice and Dectin-2KO mice were infected with *S. pneumoniae.* Cytokine concentrations in BALF were measured 12 h post-infection. Each group consists of five to seven mice. Similar results were obtained in three independent experiments. *, *p < 0.05*; *NS*, not significantly different

To clarify the role of Dectin-2 in the host response to *S. pneumoniae* infection, we compared the production of proinflammatory cytokines and chemokines, such as IL-1β, TNF-α, IL-6, IFN-γ, IL-17A, and MIP-2, in BALF between WT and Dectin-2KO mice 12 h after infection. As shown in Fig. 4, the production of IFN-γ was significantly attenuated in Dectin-2KO mice compared with WT mice, although there was no significant difference in the production of other cytokines and chemokines.

**Fig. 5:**
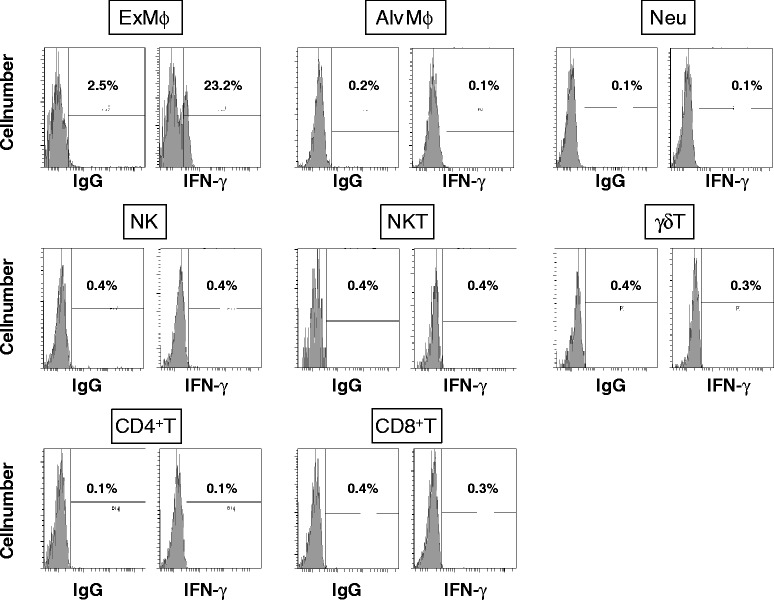
Cellular source of IFN-γ production in the lungs after pneumococcal infection. WT mice were infected with *S. pneumoniae.* The lung leukocytes prepared at 12 h post-infection were stained with various mAbs to identify populations of myeloid cells and lymphocytes, and intracellular IFN-γ expression was analyzed in each population using flow cytometry. Cut-off lines were determined on the histograms stained with isotype-matched IgG. Ex Mφ, exudate macrophages; Alv Mφ, alveolar macrophages; Neu, neutrophils; IgG, isotype-matched IgG

To define the cellular source of IFN-γ production, we used flow cytometry to examine the intracellular expression of this cytokine in various cells in the lungs of WT mice 12 h after infection. IFN-γ was expressed only in exudate macrophages, but not in alveolar macrophages, neutrophils, natural killer (NK) cells, NKT cells, γδT cells, CD4^+^ T cells or CD8^+^ T cells (Fig. 5).

### Reduced production of PPS3-specific Ab in Dectin-2KO mice

**Fig. 6:**
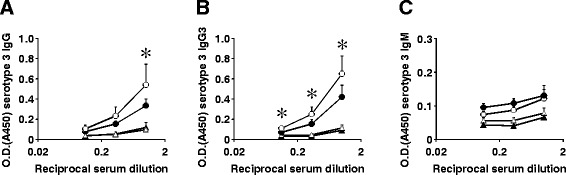
Reduced production of serotype-specific Ab against pneumococcal capsular polysaccharides in Dectin2KO mice. Anti-PPS3 IgG (**a**), IgG3 (**b**) and IgM (**c**) concentrations in BALF before infection or 24 h post-infection were measured as OD450 values at × 1, ×3 and × 9 dilution, respectively. Each group consists of six to seven mice. Similar results were obtained in three independent experiments. *, *p < 0.05*, compared with Dectin-2KO mice 24 h post-infection. Open circles, WT mice 24 h post-infection; closed circles, Dectin-2KO mice 24 h after infection; open triangles, WT mice before infection; closed triangles, Dectin-2KO mice before infection

Serotype-specific IgG against capsular polysaccharides plays a critical role as an opsonin in the phagocytosis of *S. pneumoniae* by neutrophils, which strongly promotes its eradication [17]. IgG3 is a major subclass of IgG produced under the stimulation of TI-2 Ags such as pneumococcal capsular polysaccharides [18]. We next measured PPS3-specific IgG and IgG3 levels in BALF 24 h after infection. As shown in Fig. 6a and b, the levels of PPS3-specific IgG and IgG3 in BALF were significantly increased 24 h after infection with *S. pneumoniae* in both WT and Dectin-2KO mice, and their levels were significantly lower in Dectin-2KO mice compared with WT mice. By contrast, PPS3-specific IgM levels in BALF were low, and there was no difference in the levels between WT and Dectin-2KO mice (Fig. 6c).

### Role of Dectin-2 in the activation of dendritic cells upon stimulation with *S. pneumoniae*

**Fig. 7:**
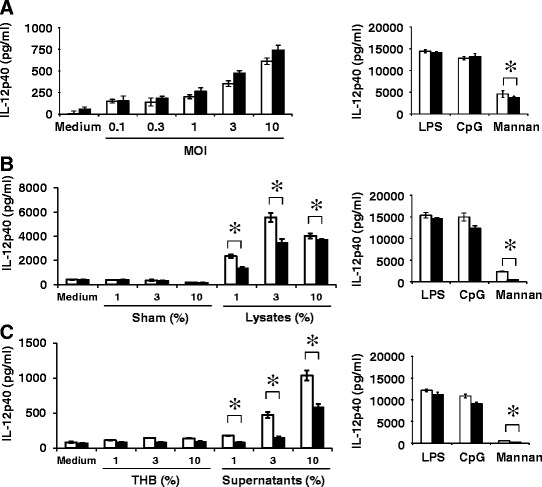
Effect of Dectin-2 deficiency on IL-12p40 production by BD-DCs upon stimulation with *S. pneumoniae*. BM-DCs from WT mice (open columns) or Dectin-2KO mice (solid columns) were cultured with live *S. pneumoniae* (**a**); lysates from *S. pneumoniae* or sham-operated PBS (Sham) (**b**); *S. pneumoniae* culture supernatant or Todd-Hewitt broth (THB) (**c**); or LPS (1 μg/ml), CpG (1 μg/ml) or mannan (3 mg/ml) for 24 h. IL-12p40 concentrations in the culture supernatants were measured using ELISA. Each column shows the mean ± SD of triplicate culture. Similar results were obtained in three independent experiments. MOI, multiplicity of infection. *, *p < 0.05*; *NS*, not significantly different

To address the role of Dectin-2 in the cellular response to *S. pneumoniae*, we examined how the lack of Dectin-2 affected the production of IL-12p40 by bone marrow-derived dendritic cells (BM-DCs) upon stimulation with viable *S. pneumoniae*, lysates or culture supernatant from this bacterium. As shown in Fig. 7a, IL-12p40 production by BM-DCs was almost comparable between WT and Dectin-2 KO mice, when stimulated with viable pneumococcus, whereas IL-12p40 was not produced by BM-DCs stimulated with heat-killed bacteria (data not shown). By contrast, IL-12p40 synthesis by BM-DCs was significantly reduced in Dectin-2KO mice compared with WT mice, when stimulated with *S. pneumoniae* lysates at lower doses (Fig. 7b). Similar results were obtained when BM-DCs were stimulated with *S. pneumoniae* culture supernatant (Fig. 7c).

**Fig. 8:**
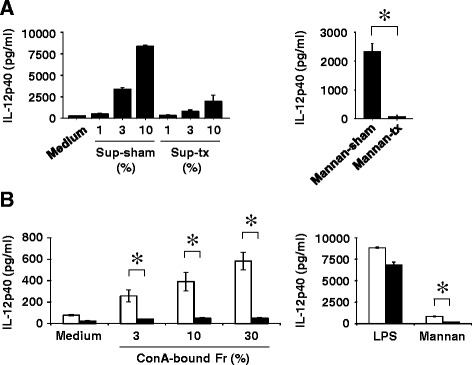
Dectin-2-dependent IL-12p40 production by BM-DCs in the ConA-bound fraction of *S. pneumoniae* culture supernatant. **a** BM-DCs derived from WT mice were stimulated with the ConA-Sepharose4B-treated or sham-treated supernatant from *S. pneumoniae* or mannan for 24 h. **b** BM-DCs from WT mice (open columns) or Dectin-2KO mice (solid columns) were cultured with the ConA-Sepharose4B-bound fraction of *S. pneumoniae* culture supernatants, LPS (1 μg/ml) or mannan (3 mg/ml) for 24 h. The IL-12p40 concentration in the culture supernatants was measured by ELISA. Each column shows the mean ± SD of triplicate culture. Similar results were obtained in three independent experiments. Sup-sham, sham-treated supernatants; Sup-tx, ConA-Sepharose4B-treated supernatants; Mannan-sham, sham-treated mannan; Mannan-tx, ConA-Sepharose4B-treated mannan; ConA-bound Fr, CpnA-Sepharose4B-bound fraction. * *p < 0.05*

To determine which *S. pneumoniae* molecule is recognized by Dectin-2, we examined how depletion of the Concanavalin A (ConA)-bound fraction in culture supernatants affects IL-12p40 synthesis by BM-DCs. IL-12p40 synthesis by BM-DCs from WT mice was abolished when stimulated with the culture supernatant that was depleted of the ConA-bound fraction (Fig. 8a). In further experiments, we examined whether the ConA-bound fraction in culture supernatant stimulated BM-DCs and whether this activity was dependent on Dectin-2. As shown in Fig. 8b, the ConA-bound fraction induced IL-12p40 production by BM-DCs from WT mice and this activity was completely abrogated in BM-DCs from Dectin-2KO mice, similar to the response caused by mannan.

## Discussion

In the present study, we evaluated the role of Dectin-2 in the neutrophil-mediated host defense to pneumococcal infection. Our data indicate that a defect in Dectin-2 rendered mice highly susceptible to a serotype 3 strain of *S. pneumoniae*, as shown by elevated mortality and an increased bacterial burden in the lungs. It is well documented that neutrophil-predominant inflammatory responses play a pivotal role in eradicating this bacterium [3]. Therefore, we predicted that a defect of Dectin-2 expression impaired the recruitment of neutrophils and clearance of *S. pneumoniae* in the lungs after infection. Dessing and co-workers previously demonstrated that TLR2- and TLR4-mediated recognition of pneumococcal components induced the production of proinflammatory cytokines, such as IL-6 and IL-1β, and chemokines, such as MIP-2 and KC, which are critical for neutrophil accumulation in inflamed tissues [19, 20]. They also found that a lack of TLR2 led to earlier death from pneumococcal meningitis [21]. In the present study, however, neutrophil accumulation and TNF-α, IL-1β, IL-17A and MIP-2 synthesis in the lungs after infection with *S. pneumoniae* were not significantly different between WT and Dectin-2KO mice. By contrast, the engulfment of pneumococcus by neutrophils was significantly impaired in Dectin-2KO mice compared with WT mice. These results suggest that Dectin-2 may be involved in the phagocytic killing of *S. pneumoniae* by neutrophils rather than in the accumulation of these cells. Recently, a similar observation was reported by Albiger et al., which addressed the role of TLR9 in the host defense against *S. pneumoniae* infection [22]. TLR9KO mice were significantly more susceptible to this infection than WT mice, probably as a result of the impaired phagocytic killing of this bacterium by macrophages.

Earlier observations demonstrated that the prognosis of bacteremia following pneumococcal pneumonia was improved in the early phase of infection, which might be related to the development of specific anti-capsular Ab [23]. Opsonization of this bacterium by IgG specific to the capsular polysaccharides is a key step in the neutrophil-mediated host defense against this infection [17, 24, 25]. TI-2 Ag, including pneumococcal capsular polysaccharides, typically elicits a rapid extrafollicular IgG response with limited isotype class switching from IgM to IgG, affinity maturation of immunoglobulin and memory B cell response [26]. However, our recent study demonstrated that the serum levels of serotype-specific IgG against pneumococcal polysaccharide Ag were increased in mice immunized with PPV [5]. Similar observations were reported by other investigators [27, 28]. Pneumococcal capsular polysaccharides have been shown to produce serotype-specific IgG, irrespective of their features as TI-2 Ag. In a clinical study, Verbinnen and co-workers demonstrated that B-1-like B cells producing serotype-specific IgG were significantly increased in the peripheral blood of healthy volunteers as early as day 5 after immunization with PPV [29]. In addition, Haas and co-workers demonstrated that B-1 B cells were involved in the protection during early responses against *S. pneumoniae* infection through the synthesis of IgG3 to TI-2 Ag [30]. Although B cell subsets producing IgG3 remain to be examined, the present data demonstrated that Dectin-2 was involved in the production of serotype-specific IgG3 in BALF as early as 24 h after infection was significantly reduced in Dectin-2KO mice compared with WT mice.

Earlier investigations reported the involvement of IFN-γ in isotype class switching of IgM to IgG3 in B cells upon stimulation with TI-2 Ag [31], although in the current study, it remains unclear whether IgG3 was produced as a result of class-switching from IgM because of equivalent anti-PPS3 IgM in BALF between WT and Dectin-2KO mice. In addition, Marchi and co-workers demonstrated that IFN-γ enhanced opsonized zymosan phagocytosis and ROS release by neutrophils [32]. Recently, we reported that a defect in Dectin-2 led to reduced IFN-γ production by NKT cells during immunization with PPV [5]. Similarly, in the present study, IFN-γ synthesis in the lungs was significantly attenuated in Dectin-2KO mice compared with WT mice after infection with *S. pneumoniae*. Thus, IFN-γ may play a pivotal role in regulating the phagocytic killing by neutrophils as a downstream event in Dectin-2-mediated recognition of pneumococcal capsular polysaccharides. Previously, we demonstrated that IL-12 plays an important role in host defense against pneumococcal infection by promoting the production of IFN-γ [13]. In the present study, Dectin-2 was essential for triggering IL-12p40 production by DCs upon simulation with *S. pneumoniae* culture supernatant, which supports the above hypothesis.

The ConA-bound fraction in *S. pneumoniae* culture supernatant induced IL-12p40 production by BM-DCs, and this activity was completely abrogated when BM-DCs were derived from Dectin-2KO mice. These results suggest that certain ConA-bound moieties of the capsular polysaccharides may be involved in Dectin-2-mediated recognition of *S. pneumoniae*. In earlier studies, Lee and co-workers demonstrated that Dectin-2 had an ability to bind Glc-, Gal-, GlcNAc- and GalNAc-BSA, in addition to the usual Man- and Fuc-BSA [33]. Additionally, ConA has been known to bind molecules that contain α-D-mannose, α-D-glucose and sterically related residues with available C-3, C-4 or C-5 hydroxyl groups [34]. These previous findings suggest that some glucosyl residue in the capsular polysaccharides might be recognized by Dectin-2. Further investigations are necessary to define the precise polysaccharide structure that contributes to *S. pneumoniae* recognition through Dectin-2.

Among different *S. pneumoniae* serotypes, capsular polysaccharide structures are not identical [35]. McGreal and co-workers previously reported that serotype 3 capsular polysaccharide inhibited the interaction between Dectin-2 and mannans, whereas other serotypes, such as 2, 9 V, 14, 18C and 19 F, did not show such an effect [36], suggesting a distinct role for Dectin-2 in the recognition of different *S. pneumoniae* serotypes. In the present study, we used only a serotype 3 strain of *S. pneumoniae*, and therefore, these findings may not be generalizable to all pneumococcal infections.

## Conclusions

In the present study, we demonstrate that Dectin-2KO mice were more susceptible to infection with a serotype 3 strain of *S. pneumoniae* than WT mice, as shown by a shorter survival time, larger bacterial burden and lower IFN-γ production in the lungs of Dectin-2KO mice. Our results suggest that Dectin-2-dependent IL-12 production may contribute to IFN-γ synthesis and subsequent production of serotype-specific anti-capsular polysaccharide IgG after *S. pneumoniae* serotype 3 infection, which may promote opsonization of this bacterium for engulfment. Thus, the present study may provide important implications for better understanding in the host defense mechanism against *S. pneumoniae* serotype 3 and for developing more effective vaccine strategies against this infection.

## Methods

### Mice

Dectin-2KO mice were generated by homologous recombination of the *Clec4n* gene as described previously [7]. WT littermate mice of the Dectin-2KO mice were used as controls. Male or female mice at 6 to 8 weeks of age were used for the experiments. The mice were bred under specific pathogen-free conditions at the Animal Facility, Tohoku University Graduate School of Medicine (Sendai, Japan). All experimental procedures involving animals followed the Regulations for Animal Experiments and Related Activities at Tohoku University and were approved by the ethics committees of Tohoku University.

### Bacteria

A serotype-3 clinical strain of *S. pneumoniae*, designated as URF918, was established from a patient with pneumococcal pneumonia [37]. The bacteria were cultured in Todd-Hewitt broth (Difco, Detroit, MI, USA) at 37 °C in a 5 % CO_2_ incubator, harvested at the mid-log phase of growth and then washed twice in phosphate buffered saline (PBS). The inoculum was stored at −80 °C until use.

### *S. pneumoniae* infection

WT or Dectin-2KO mice were anaesthetized by an intraperitoneal injection of 70 mg/kg pentobarbital (Abbott Laboratory, North Chicago, IL, USA) and restrained on a small board. Live *S. pneumoniae* (0.75-3 × 10^5^ colony forming units (CFU)) at 50 μl per mouse were inoculated by insertion of a 24G intravenous (IV) catheter (Terumo, Tokyo, Japan) into the trachea. Colony counts were performed to confirm the accuracy of inoculum CFU as a means to determine CFU/ml for *S. pneumoniae* using a 5 % sheep blood tryptic soy agar plate (Nissui Pharmaceutical Co., Ltd., Tokyo, Japan).

### Enumeration of viable *S. pneumoniae*

WT and Dectin-2KO mice were sacrificed on day 3 post-infection, and the lungs were dissected carefully and excised. They were then homogenized in 5 ml of PBS by teasing with stainless mesh at room temperature. The homogenates (100 μl) were diluted in twofold series using sterile half saline and inoculated onto a 5 % sheep blood tryptic soy agar plate (Nissui Pharmaceutical Co., Ltd.). The homogenate was then cultured for 24 h at 37 °C in 5 % CO_2_, and the number of colonies was counted.

### Lung histology

Lungs were isolated from WT or Dectin-2KO mice 12 h after pneumococcal infection, and fixed in 10 % buffer formalin, dehydrated and embedded in paraffin. Sections were cut and stained with hematoxylin-eosin (H-E) or Gram stain at the Biomedical Research Core, Animal Pathology Platform of Tohoku University Graduate School of Medicine (Sendai, Japan).

### Preparation of BALF

BALF samples from WT or Dectin-2KO mice were collected as described below. Briefly, after bleeding under anesthesia with isoflurane, the chest was opened and the trachea was cannulated with the outer sheath of a 22G IV catheter/needle unit (Terumo), followed by lung lavage three times with 1 ml of chilled PBS. Then, 1 × 10^5^ cells were centrifuged onto a glass slide using StatSpin Cytofuge 2 (Iris Sample Processing, Franklin, MA, USA), and then stained with Diff-Quick or Gram stain. After centrifugation of BALF, supernatants were stored at −80 °C for cytokine assay. To analyze the leukocyte fraction and *S. pneumoniae* phagocytosis by neutrophils, at least 500 cells were examined using light microscopy.

### Measurement of cytokine concentration

TNF-α and IFN-γ levels in the BAL fluids, and IL-12p40 levels in culture supernatants were determined by enzyme-linked immunosorbent assay (ELISA) using capture and biotinylated developing antibodies (BD Biosciences, Franklin Lakes, NJ, USA). Collected BALF was assessed using ELISA kits for IL-1β, IL-6, IL-17A (eBioscience, San Diego, CA, USA) and MIP-2 (RayBiotech, Norcross, GA, USA). The detection limits of assays for TNF-α, IFN-γ, IL-12p40, IL-1β, IL-6, IL-17A and MIP-2 were 50, 15, 15, 8, 4, 4 and 2.74 pg/ml, respectively.

### In vivo neutrophil phagocytosis

Twelve hours after pneumococcal infection, WT and Dectin-2KO mice were sacrificed and BALF was collected. The cells were spun onto glass slides, and the phagocytic index and phagocytic rate were examined by light microscopy following Gram staining. The phagocytic rate was calculated as follows: (number of neutrophils engulfing pneumococcus/total number of neutrophils) × 100. The phagocytic index was calculated as follows: (number of phagocytized pneumococcus/total number of neutrophils engulfing pneumococcus).

### Measurement of serotype-specific Antibodies

BALF samples were collected before infection or 24 h post-pneumococcal infection. The quantities of serotype-specific antibodies (Ab) against pneumococcal polysaccharide type 3 (PPS3) in BALF were measured by ELISA. Microtiter plates (Nunc A/S, Roskilde, Denmark) were coated with 3 μg/ml of PPS3 (American Type Culture Collection, Manassas, VA, USA) in PBS for 1 h at 37 °C. Before testing, serum samples were diluted with 0.05 % skim milk PBS. HRP-conjugated goat anti-mouse IgG, IgG3 or IgM antibodies (Southern Biotechnology Associates, Birmingham, AL, USA) diluted with 1:4000 were used as detection Ab. The concentrations of IgG, IgG3 and IgM were determined based on the absorbance at 450 nm.

### Analysis of intracellular IFN-γ expression

Lung leukocytes, prepared as previously described [38], were stained with APC-anti-CD3ε mAb (clone 145-2C11, Biolegend, San Diego, CA, USA), Pacific Blue-anti-CD4 mAb (clone GK1.5, Biolegend), APC/Cy7-anti-CD8 mAb (clone 53–6.7, Biolegend), PE/Cy7-anti-NK1.1 mAb (clone PK136, Biolegend) and PE-anti-TCRδ mAb (clone GL3, Biolegend). The cells were also stained with PE-anti-CD11b mAb (clone M1/70, Biolegend), PE/Cy7-anti-Gr-1 mAb (clone RB6-8C5, Biolegend) and Pacific Blue-anti-F4/80 mAb (clone BM8, Biolegend). After washing twice, the cells were incubated in the presence of cytofix/cytoperm (BD Biosciences), washed twice in BD perm/wash solution (BD Biosciences) and stained with FITC-anti-IFN-γ mAb (clone XMG1.2, Biolegend). Isotype-matched IgG was used for control staining. The stained cells were analyzed using a BD FACS Canto^TM^ II flow cytometer (BD Biosciences). Data were collected from 20,000 to 30,000 individual cells using forward-scatter and side-scatter parameters to set a gate on the lymphocyte or myeloid cell population.

### Preparation and culture of dendritic cells

Bone marrow cells from WT mice and Dectin-2KO mice were cultured at 2 × 10^5^/ml in 10 ml RPMI1640 medium (Nippro, Osaka, Japan) supplemented with 10 % fetal calf serum (FCS) (Biowest, Nuaillé, France), 100 U/ml penicillin G, 100 μg/ml streptomycin, 2 mM L-glutamine and 50 μM 2-mercaptoethanol (Sigma-Aldrich, St. Louis, MO, USA) containing 20 ng/ml murine granulocyte-macrophage colony-stimulating factor (GM-CSF; Wako Pure Chemical Industries, Ltd., Osaka, Japan). On day 3, 10 ml of the same medium was added, followed by replacement of half of the medium with GM-CSF-containing culture medium on day 6. On day 8 or 9, non-adherent cells were collected and used as BM-DCs. BM-DCs were cultured at 1 × 10^5^/ml with various stimuli at 37 °C in a 5 % CO_2_ incubator for 24 h. Lipopolysaccharide (LPS) prepared from *Escherichia coli* O-111 (Sigma-Aldrich), phosphorothioated CpG1826 oligonucleotide (Hokkaido System Science, Sapporo, Japan), and mannan from *Saccharomyces cerevisiae* (Sigma-Aldrich) were used as controls.

### Preparation of *S. pneumoniae* homogenates

*S. pneumoniae* were grown on 5 % sheep blood tryptic soy agar plate (Nissui Pharmaceutical Co., Ltd., Tokyo, Japan) at 37 °C in a 5 % CO_2_ incubator for 24 h. Harvested colonies were crushed in PBS using 0.1 mm glass beads and a Multi-Beads Shocker (Yasuikikai, Osaka, Japan) at 2500 rpm and 4 °C for 40 cycles (30 s on/30 s off), passed through a 40 μm nylon mesh filter (Becton, Dickinson and Company, Franklin Lakes, NJ, USA), and then stored at −80 °C until use. Sham-operated PBS were treated identically without *S. pneumoniae* and used as controls.

### Preparation of *S. pneumoniae* culture supernatants

*S. pneumoniae* was diluted with half saline until the turbidity reached 0.5 using the McFarland standard, inoculated in 19 volumes of Todd-Hewitt broth (Difco) and then incubated on an orbital shaker (150 rpm) at 37 °C in a 5 % CO_2_ incubator for 24 h. The culture supernatants were centrifuged, passed through a 0.45 μm membrane filter (Sartorius, Göttingen, Germany) and stored at −80 °C until use. Todd-Hewitt broth incubated without *S. pneumoniae* was used as a control.

### ConA-affinity chromatography of *S. pneumoniae* culture supernatants

ConA-Sepharose4B (GE Healthcare Bio-Sciences AB, Uppsala, Sweden) was prepared according to the manufacturer’s instructions. Briefly, a polystyrene column (0.6 × 18 cm; bed volume, 5 ml) was packed with ConA-Sepharose4B. The column was washed with 50 ml of binding buffer (20 mM Tris–HCl, 0.5 M NaCl, pH 7.4) for regeneration and re-equilibration. *S. pneumoniae* culture supernatants were applied to the column continuously at a flow rate of 3 ml/h. The bound-fractions were eluted with an elution buffer of 0.4 M methyl-α-D-mannopyranoside (Sigma-Aldrich) in 20 mM Tris–HCl at pH 7.4 and with 0.5 M NaCl, and dialyzed against PBS using a membrane with a 2-kDa molecular weight cutoff (Thermo Fisher Scientific Inc., IL, USA). All the chromatographic operations and dialysis were performed at 4 °C. To collect the ConA-unbound fraction, *S. pneumoniae* culture supernatants were incubated with ConA-Sepharose4B for 15 min at room temperature, and then the ConA-Sepharose4B beads were removed using centrifugation. Sham treatment was performed using Sepharose4B beads. Similarly, mannan received ConA-Sepharose4B or sham treatment.

### Statistical analysis

Statistical analysis was conducted using GraphPad Prism 5 software (GraphPad Software, La Jolla,CA,USA). Data are presented as the mean ± standard deviation (SD). Differences between the two groups were tested using a two-tailed analysis in an unpaired Student’s *t*-test. Survival data was analyzed using the Kaplan-Meier log rank test. A *p* value less than 0.05 was considered significant.

## Abbreviations

CLRs: C-type lectin receptors
KO: Knock-out
WT: Wild type
BALF: Bronchoalveolar lavage fluid
BM-DCs: Bone marrow-derived dendritic cells
OPK: Opsonophagocytic killing
ROS: Reactive oxygen species
TI-2: Thymus-independent type 2
PPV: Pneumococcal polysaccharide vaccine
MIP: Macrophage inflammatory protein
KC: Keratinocyte-derived chemokine
PPRs: Pattern recognition receptors
PPS3: Pneumococcal polysaccharide type 3
FCS: Fetal calf serum
LPS: Lipopolysaccharide
ConA: Concanavalin A
